# Frequency of Electrolyte Derangement after Transurethral Resection of Prostate: Need for Postoperative Electrolyte Monitoring

**DOI:** 10.1155/2015/415735

**Published:** 2015-05-18

**Authors:** Wajahat Aziz, M. Hammad Ather

**Affiliations:** Section of Urology, Department of Surgery, Aga Khan University, P.O. Box 3500, Stadium Road, Karachi 74800, Pakistan

## Abstract

*Objective*. To determine the electrolyte derangement following transurethral resection of prostate (TURP).* Methods*. All patients undergoing TURP from June 2012 to April 2013 were included. Preoperative electrolytes were performed within a week of procedures. Monopolar TURP using 1.5% glycine was performed. Serum Na^+^ and K^+^ were assessed within 1 hour postoperatively and subsequently if clinically indicated.* Results*. The study included 280 patients. Sixty-six patients (23.6%) had electrolyte derangement after TURP. Patients with deranged electrolytes were older (mean age of 73.41 ± 4.08 yrs. versus 68.93 yrs. ± 10.34) and had a longer mean resection time (42.5 ± 20.04 min versus 28.34 ± 14.64 min). Mean weight of tissue resected (41.49 ± 34.46 g versus 15.33 ± 9.74 g) and volume of irrigant used (23.55 ± 15.20 L versus 12.81 ± 7.57 L) were also significantly higher in patients with deranged electrolytes (all *p* = 0.00). On multivariate logistic regression analysis preoperative sodium level was found to be a significant predictor of postoperative electrolyte derangement (odds ratio 0.267, S.E. = 0.376, and *p* value = 0.00).* Conclusion*. Electrolyte derangement occurs in older patients, with larger amount of tissue and longer time of resection and higher volume of irrigant, and in those with lower serum preoperative sodium levels.

## 1. Introduction

Transurethral resection of prostate (TURP) is one of the most common urological procedures performed. Despite introduction of several minimally invasive options like Holmium Laser Enucleation and Holmium Laser Ablation, TURP is still considered the gold standard for surgical management of Benign Prostatic Obstruction (BPO) [1].

Complications after TURP are frequent [2]. Early complications of TURP include bleeding, sepsis, TUR syndrome, incontinence, and urinary retention. The incidence of early complications of TURP has decreased considerably over the past few decades. This is largely attributable to standardization of the procedure, better perioperative management [3], and better anesthetic techniques [4]. Bleeding requiring transfusion, acute kidney injury, and transurethral resection syndrome are the complications of TURP in early postoperative period that greatly influence morbidity of the procedure and may even lead to mortality [5].

Electrolyte imbalance is one of the most worrisome complications of TURP especially due to risk of developing overt TUR syndrome. This syndrome results from the absorption of irrigating fluid through prostatic veins exposed by breaches in the prostatic capsule during TURP. The irrigation fluid used during resection is absorbed via these channels and leads to hypervolemic hyponatremia. Mental confusion, bradycardia, hypotension/hypertension, nausea, vomiting, and visual disturbances associated with hyponatremia are most commonly observed symptoms [6]. These symptoms are mostly the result of brain edema caused by hypervolemic hyponatremic state. Hyperkalemia can also occur after TURP attributable mainly to cell lysis and release of intracellular potassium. Acute kidney injury secondary to obstruction or sepsis can also lead to hyperkalemia in some cases [7].

TUR syndrome has become a rare event in recent years with better appreciation of pathophysiology and advances in technology. Several modifications have led to decreased incidence of this complication. Among these are development of continuous flow resectoscopes, utilization of “nonhemolytic” solutions such as glycine, sorbitol, and mannitol, use of bipolar circuitry, and advances in training techniques [8]. TUR syndrome was found in only 1% of patients in a recent multicenter study [9]. Certain risk factors are known to be associated with increased risk of TUR syndrome including volume and type of irrigant used, resection time, weight of tissue resected, and use of monopolar diathermy [10].

Monitoring of serum electrolytes after TURP is variable in different centers and does not usually take into account the risk factors for developing electrolyte derangement. Major urological associations do not have specific guidelines regarding post-TURP electrolyte monitoring, although such testing is performed routinely in many centers. Routine electrolyte measurement in all patients undergoing TURP irrespective of risk factors for developing TURP syndrome is burdensome for both patient and hospital staff and also incur additional cost. The purpose of the present study is to evaluate the frequency and risk factors for electrolyte derangement after TURP.

## 2. Material and Methods

Cross-sectional study was conducted at inpatient units of a university hospital. All patients above age 50 years and above undergoing TURP from June 2012 to April 2013 were included. Patients who refuse to participate in the study, who received diuretic intraoperatively or are in immediate postoperative period, and patients with already deranged electrolytes (as per operational definition) or raised serum creatinine (i.e., serum creatinine >1.2) were excluded. Ethical review committee approval was taken before commencement of work on the subject. Preoperative electrolytes done within 1 week before the procedure were recorded. Any one of the six urology consultants carried out TURP or resident under supervision of these consultants using a continuous flow resectoscope with monopolar diathermy and 1.5% glycine as an irrigant. A 3 cc postoperative serum sample was taken within 1 hour of the end of procedure for electrolytes assessment. Serum sodium and potassium were measured using ion selective electrode method. Operative parameters, resection time, volume of irrigant use, weight of tissue resected, volume and type of intravenous fluids, and postoperative clinical symptoms, were recorded. Deranged electrolytes were defined as presence of any or both of serum sodium <130 or >145 mmol/L and serum potassium <3.5 or >5.5 mmol/L.

Data was analyzed using SPSS version 17.0. Results were described in terms of mean and standard deviation for continuous variables. Categorical variables were described in terms of frequency and percentage. The proportion of deranged sodium, potassium, and creatinine after TURP was calculated. Stratification was done with respect to comorbid, current smoking status, and surgeon. Chi square test and Fischer's exact test were applied to determine significance where appropriate. Continuous variables like age, preoperative electrolytes, resection time, volume of irrigation fluid used, and weight of tissue resected were compared using *t*-test. *p* value of less than 0.05 was considered significant. Binary Logistic regression analysis was done to identify risk factors of electrolyte derangement.

## 3. Results

Two hundred and eighty consecutive patients who underwent TURP were included in the study (Table 1). Mean age of patient was 69.98 years (range 51–90, S.D. 9.45). When patients with electrolyte derangement were compared with those having no electrolyte derangement, the former group was found to be significantly older and had a mean resection time significantly higher than those with no electrolyte derangement. The mean weight of tissue resected and the volume of irrigant used were also significantly higher in patients with postoperative electrolyte derangement (Table 2).

Comorbids including ischemic heart disease (IHD), congestive cardiac failure (CCF), and diabetes mellitus (DM) were not found to be associated with postoperative electrolyte derangement. Hypertensive patients had a higher proportion of electrolyte derangement compared to normotensive (Table 3).

On multivariate logistic regression analysis the only significant factor predicting postoperative electrolyte derangement was preoperative sodium level after controlling for resection time, volume of irrigant used, weight of tissue resected, age, and hypertension (Table 4).

### 3.1. Discussion

Transurethral resection syndrome (TUR syndrome) is caused by fluid absorption from venous channels in prostatic bed in the presence of continuous irrigation. Absorption of this fluid leads to changes in serum electrolytes and potentially can lead to clinical TUR syndrome. In our study we found a decrease in serum concentration of both sodium and potassium postoperatively. Although the rate of fluid absorption during TURP depends upon a number of factors (Table 5), the average rate is 20 mL/min [11]. We identified an overall decrease in the serum levels of both sodium and potassium, though the overall magnitude of this decrease is subtle (mean decrease of 3.13 mEq/L for sodium and mean decrease of 0.082 for potassium).

Hyperkalemia following TURP is partly explained by cell lysis as happened during resection of tissue. Absorption of fluid into circulation is an alternate mechanism that can cause hyperkalemia after TURP [12]. ECG changes and cardiac toxicity caused by hyperkalemia usually occur at serum levels above 6 mEq/L. We did not encounter significant rise of serum potassium postoperatively in our series. This is partially explained by hemodilution caused by fluid absorption to offset any changes caused by hemolysis. Also with 1.5% glycine as irrigant, hemolysis is minimal as compared to other hypoosmolar irrigants like water. Singhania and colleagues compared monopolar versus bipolar saline resection in 60 patients and found no significant changes in potassium levels in either group [13].

When patients with deranged electrolytes were compared with those having no electrolyte derangement, a number of important findings were noted.

First, patients with electrolyte derangement were significantly older than those without electrolyte derangement. Uchida et al. found age of the patient undergoing TURP as a significant risk factor for perioperative blood transfusion and attributed it to more rigid vasculature in elderly, which allows for persistent opening of venous channels [14]. The same mechanism can account for increased fluid absorption and electrolyte derangement in elderly patients.

Second, mean weight of tissue resected was found to be higher in those patients undergoing TURP. The amount of fluid absorption depends mainly on the number and size of venous sinuses opened [15]. The weight of tissue resected serves as a surrogate marker for the number of venous sinuses opened in prostatic bed. To decrease the likelihood of TUR syndrome, European Urology Association suggests open surgery or transurethral holmium laser enucleation for men with prostates >80 mL [16], whereas TURP is considered the standard procedure for men with prostate 30–80 mL.

Third, increased resection time correlates with electrolyte derangement in our study. Provided that the irrigation fluid column is kept at a constant height, a constant volume of fluid is obtained per minute during resection. However, the amount of fluid absorption not only depends on the duration of exposure of the exposed venous sinuses to the irrigating fluid but also upon the number of prostatic venous sinuses opened and hydrostatic pressure at the prostatic bed. Madsen and Naber demonstrated that hydrostatic pressure at the prostatic bed is an important factor determining fluid absorption during TURP. This hydrostatic pressure depends upon the height of irrigating fluid column and pressure inside bladder during surgery [17]. The ideal height of irrigating fluid is suggested to be 60 cm so that approximately 300 mL of fluid is obtained per minute during resection to maintain good vision. For our study, we fixed the irrigation fluid column height so that other determinants can be assessed. In order to limit the likelihood of a serious electrolyte derangement, it is advocated that resection times should be limited to 1 hour.

Fourth, volume of irrigant used was found to be significantly higher in patients with deranged electrolytes. We used 1.5% glycine in all patients undergoing TURP. So, the type of irrigant used is not a factor in determining fluid absorption in our patients. Volume of irrigant used is consistently found to correlate with the risk of postoperative electrolyte derangement in previous studies [18].

Finally, hypertensive patients were found to be at higher risk of developing postoperative electrolyte derangement. Some antihypertensives, for example, angiotensin converting enzyme inhibitors, are known to inhibit normal regulation of fluid balance and may even cause hyponatremia [19]. On the other hand although preoperative diuretic use was found to be more common in patients with electrolyte derangement, a conclusive statement cannot be made due to the small number of patients using diuretics preoperatively.

Our sample size calculation was based upon frequency of electrolyte derangement, so logistic regression analysis was not part of initial study protocol. However, on logistic regression analysis, the most significant factor predicting electrolyte derangement was preoperative sodium level with Exp(*B*) of 0.267; that is, for each unit rise in preoperative sodium level the odds of electrolyte derangement decrease by approximately 27%. This is an important finding and suggests that low normal values of serum sodium should alert the surgeon to the possibility of postoperative electrolyte derangement.

Few studies have investigated the usefulness of routine electrolyte testing following TURP. Most of them focused on post-TURP Hb monitoring [20]. Emphasis on preoperative optimization and better operative techniques has made transfusion during TURP a rare event. In our study none of the patients had preoperative blood transfusion, so its correlation with electrolyte derangement immediately after TURP could not be assessed. Hakem et al. retrospectively studied 137 patients; they found low postoperative sodium in 2 patients, but there was no TUR syndrome in any patient [7]. They concluded that routine postoperative blood testing following TURP is not required in all cases and recommended blood testing based on clinical need or following technically demanding operations.

Overall 66/280 patients had deranged electrolytes after TURP in our study. None of the patients had clinical TUR syndrome. The significance of mild hyponatremia after TURP is unknown. It may contribute to postoperative nausea, vomiting, and delayed recovery from general anesthesia in at least some patients. In a cross-sectional study patients undergoing TURP, hyponatremia was observed in 28 out of 40 (70%) patients. In this study a value of <135 mEq/L was used to define hyponatremia [6]. Although our clinical laboratory also uses same value as a lower range for normal sodium, we used a threshold of <130 mEq/L as it is more clinically relevant [21].

Several strategies have been proposed to reduce the risk of fluid absorption during TURP, but none is capable of eliminating this complication altogether. It has been suggested to keep resection time below 60 min to minimize fluid absorption; TUR syndrome has been reported after a resection time of only 15 min [22].

Monitoring the extent of fluid absorption during surgery has been suggested to control fluid balance in every patient. The most viable methods to monitor fluid absorption are ethanol monitoring and gravimetric weighing [23]. Newer techniques, such as bipolar resectoscopes and vaporizing the tissue instead of resecting tissue, have reduced fluid absorption and its consequent electrolyte derangement, so routine monitoring of fluid absorption has been largely abandoned outside a study setting. However there is no consensus on routine monitoring of postoperative electrolytes. It has been suggested that with improvements in technology and use of isotonic, nonhemolytic solutions; electrolyte derangement is rare. Particularly with the use of isotonic saline and bipolar resection TURP syndrome is of historical interest only [24].

In light of our findings we suggest a more realistic approach. Electrolyte derangement occurs commonly in patients undergoing TURP, as manifested by a frequency of 16% in our series, though full-blown TUR syndrome is rare. Electrolyte monitoring should be considered in patients having risk factors for increased fluid absorption.

### 3.2. Conclusion

Electrolyte derangement after TURP is not uncommon. The need for monitoring electrolyte following TURP should be individualized, taking into account the weight of resected tissue, volume of irrigation used, resection time, increasing age, and hypertension. Low normal values of serum sodium should alert the surgeon to the possibility of postoperative electrolyte derangement.

## Figures and Tables

**Table 1:** Baseline characteristics, *n* = 280.

	Median	Std. deviation
Age	73	9.45
Preoperative sodium	142	5.03
Preoperative potassium	4.5	0.46
Preoperative serum creatinine	0.9	0.18
Postoperative sodium	138	5.43
Postoperative potassium	4.3	0.49
Volume of irrigant used	12	10.87
Weight of tissue resected	13	21.76
Resection time	30	17.13

**Table 2:** Comparison of continuous variables between those without electrolyte derangement and those with electrolyte derangement. *p* value calculated using Student's *t*-test.

	No electrolyte derangement *n* = 214	Electrolyte derangement *n* = 66	*p* value
Mean age (years)	68.93 ± 10.35	73.41 ± 4.08	0.00
Mean preoperative serum sodium	141.47 ± 2.70	131.33 ± 2.26	0.00
Mean preoperative serum potassium	4.40 ± 0.42	4.13 ± 0.53	0.00
Mean preoperative serum creatinine	0.9 ± 0.19	0.8 ± 0.19	0.26
Mean postoperative serum sodium	138.19 ± 2.65	128.67 ± 5.78	0.00
Mean postoperative serum potassium	4.31 ± 0.34	4.10 ± 0.81	0.002
Mean resection time (min)	28.34 ± 14.64	42.50 ± 20.04	0.00
Mean volume of irrigant used (liters)	12.81 ± 7.57	23.55 ± 15.20	0.00
Mean weight of tissue resected (grams)	15.33 ± 9.74	41.59 ± 34.45	0.00

**Table 3:** Comparison of categorical variables between those without electrolyte derangement and those with electrolyte derangement. *p* value calculated using chi square test/Fischer's exact test where applicable.

		No electrolyte derangement, *n* = 214	Electrolyte derangement, *n* = 66	*p* value
CCF	No	203	62	0.488
	Yes	11	4	

IHD	No	179	61	0.051
	Yes	35	5	

DM	No	164	55	0.249
	Yes	50	11	

HTN	No	108	4	0.00
	Yes	106	62	

Diuretic use	No	208	58	0.006
	Yes	6	8	

Smoker	No	160	49	0.932
	Yes	54	17	

Surgeon	Resident	89	24	0.449
	Consultant	125	42	

CCF: congestive cardiac failure; IHD: ischemic heart disease; HTN: hypertension.

**Table 4:** Logistic regression analysis of predictors of electrolyte derangement.

	Exp(*B*)	S.E.	*p* value
Resection time	1.013	0.082	0.873
Volume of irrigant used	0.918	0.116	0.461
Weight of tissue resected	1.249	0.124	0.073
Age	0.934	0.102	0.502
Comorbids	0.035	2.271	0.140
Preoperative sodium	0.267	0.376	0.000
Surgeon	0.103	1.837	0.217

**Table 5:** Factors increasing fluid absorption and electrolyte derangement during TURP.

Factors increasing fluid absorption during TURP	Strategies to minimize fluid absorption
Open prostatic sinuses	
(i) Weight of tissue resected (used as surrogate marker)	(i) Consider open prostatectomy or HoLEP for >80 g prostate
(ii) Capsular breech	(ii) Avoid deep resection

Lengthy resection	
Prostatic sinuses exposed for longer time	Keep resection time under 60 min

High irrigation pressure	
(i) Height of irrigation column	(i) Keep irrigation fluid at height of 60 cm
(ii) Small capacity bladder	(ii) Continuous flow resectoscope

Hypotonic irrigant	(i) Use of isotonic irrigant (ii) Bipolar diathermy
